# Elucidating intermolecular forces to improve compatibility of kraft lignin in poly(lactic acid)

**DOI:** 10.3389/fchem.2024.1347147

**Published:** 2024-02-08

**Authors:** Esakkiammal Sudha Esakkimuthu, Veerapandian Ponnuchamy, Mika H. Sipponen, David DeVallance

**Affiliations:** ^1^ InnoRenew CoE, Izola, Slovenia; ^2^ Department of Materials and Environmental Chemistry, Stockholm University, Stockholm, Sweden; ^3^ Andrej Marušič Institute, University of Primorska, Koper, Slovenia; ^4^ Wallenberg Wood Science Center, Department of Materials and Environmental Chemistry, Stockholm University, Stockholm, Sweden; ^5^ Faculty of Mathematics, Natural Sciences and Information Technologies, University of Primorska, Koper, Slovenia

**Keywords:** lignin, poly - (lactic acid), composites, molecular dynamics simulations, hydrogen bonding, mechanical properties

## Abstract

Owing to its abundant supply from renewable resources, lignin has emerged as a promising functional filler for the development of sustainable composite materials. However, achieving good interfacial compatibility between lignin and synthetic polymers, particularly poly (lactic acid) (PLA), remains a fundamental challenge. To advance the development of high-performance bio-based composites incorporating lignin and PLA, our study has scrutinized to unravel the nuances of interfacial binding interactions with the lignin and PLA composite system. Molecular level and experimental examinations were employed to decipher fundamental mechanisms governing and demonstrating the interfacial adhesion. We synthesized casted films of lignin/PLA and acetylated lignin/PLA at varying weight percentages of lignin (5%, 10%, and 20%) and comprehensively investigated their physicochemical and mechanical properties. The inclusion of acetylated lignin in the composites resulted in improved mechanical strength and Young’s modulus, while the glass transition temperature and melting point were reduced compared to neat PLA. Systematic variations in these properties revealed distinct compatibility behaviors between unmodified lignin and acetylated lignin when incorporated into PLA. Molecular dynamics (MD) simulation results elucidated that the observed changes in material properties were primarily attributed to the acetylation of lignin. Acetylated lignin exhibited lower Coulombic interaction energy and higher van der Waals forces, indicating a stronger affinity to PLA and a reduced propensity for intermolecular aggregation compared to unmodified lignin. Our findings highlight the critical role of controlling intermolecular interactions and lignin aggregation to develop PLA composites with predictable performance for new applications, such as functional packaging materials.

## 1 Introduction

Lignocellulosic biomass is a complex and highly heterogeneous composite material of polysaccharides (cellulose and hemicellulose) and aromatic biopolymer (lignin) (Cai et al., 2017). Among these components, lignin is an undervalued by-product and energy source through combustion in lignocellulosic biorefineries such as chemical pulp mills. Lignin macromolecules originate from the radical polymerization of one or more of the so-called monolignols (Schoenherr et al., 2017). A major body of research has been conducted to find ways to utilize lignin for value-added applications. One of the directions is to use lignin as a functional filler in polymer composites (Duval and Lawoko, 2014; Upton and Kasko, 2016; Wang et al., 2016).

Incorporation of lignin in the polymer matrix can enhance thermal, mechanical, UV-protective, and radical scavenging properties of the polymers such as polyethylene, polypropylene, poly (lactic acid) (PLA), and poly (ε-caprolactone) (Thakur et al., 2014). Among them, PLA is of particular interest as an industrially compostable polyester derived from biotechnologically produced lactic acid (Garlotta, 2001; Lim et al., 2008; Rasal et al., 2010). It has been applied for several applications including food packaging, single-use items, medical applications, etc., (Jem and Tan, 2020). In 2019, 190,000 tons annually of PLA were reported to be produced (Masutani and Kimura, 2014; Dubey et al., 2017; Jem and Tan, 2020). However, the selling cost of PLA is relatively high, about 5140 US$/per ton (Ratshoshi et al., 2021) and despite its appropriate mechanical strength, PLA exhibits poor UV shielding character when used in food packaging applications. These issues disfavor PLA in comparison with aromatic polymers such as polyethylene terephthalate (PET) and polystyrene (PS) (van den Oever et al., 2017).

The blending of lignin in PLA decreases the total cost and enhances the UV barrier properties (Shankar et al., 2015; Sakai et al., 2018; Rukmanikrishnan et al., 2020; Hararak et al., 2021; Črešnar et al., 2021; Makri et al., 2022). However, direct utilization of lignin as a filler in PLA often leads to poor compatibility between the contrastive polymers. An underlying reason suggested for this incompatibility is the intermolecular aggregation of lignin in the PLA polymer. Such aggregation phenomena should be minimized in order to enhance compatibility along with thermal and mechanical properties. Simultaneously, the interaction between lignin and PLA ought to be enhanced. In such instances, chemical modification of lignin, mainly acetylation has been proposed to improve compatibility and thermal stability compared to unmodified lignin in PLA(Gordobil et al., 2014; Kim et al., 2017; Li et al., 2017; Park et al., 2019; Johansson et al., 2023).

Numerous experimental investigations have centered around incorporating diverse chemical modifications of lignin, utilizing various methods to augment the compatibility of lignin within the PLA matrix (Daassi et al., 2023; Zaidi et al., 2023). The complex interplay within these lignin-based PLA composites can be enabled through the application of advanced computational modeling techniques. Through computational simulations, we aim to unravel the intermolecular forces that control the interfacial behavior of these composites. This computational exploration governs the compatibility and structural integrity of the composite system, thereby bridging the gap between experimental observations and a comprehensive mechanistic understanding.

Molecular dynamics (MD) simulation method accounts for important interactions and allow the prediction of mechanical properties. Despite their utility to understand and predicting properties of various pure PLA (Farazin and Mohammadimehr, 2022) and PLA-based composites with different materials such as hydroxyapatite, graphene, amorphous cellulose and wood cell wall constituents (Zhang et al., 2009; Zhou et al., 2013; Hasheminejad and Montazeri, 2020; Ren et al., 2021; Ren et al., 2023). It is crucial to emphasize that the existing literature lacks substantial evidence derived from the combination of experimental findings and theoretical atomistic modeling concerning lignin within the PLA matrix. To the best of our knowledge, this study marks the first time into the investigation of both lignin and acetylated lignin in conjunction with PLA through comprehensive molecular dynamics (MD) simulations.

In the present work, we have prepared lignin/PLA (LIG/PLA) and acetylated lignin/PLA (aLIG/PLA) composites using a casting method with three different weight percentages of lignin (5%, 10%, and 20%) in the PLA matrix. The composites were systematically characterized for mechanical (tensile strength, Young’s modulus) and thermal (glass transition temperature) properties. Furthermore, the MD approach was employed to investigate the composites to provide significant insights into the intermolecular interactions. The MD simulations provided means to decipher the experimental results based on the physicochemical, dynamic, and mechanical properties such as radial distribution function, hydrogen bonding, mean square displacement, Young’s modulus, and glass transition temperature. Overall, the combination of experimental and modelling methods provided a comprehensive view of the features that determine the extent that lignin aggregates in PLA composites.

## 2 Materials and methods

Kraft softwood lignin (UPM BioPiva 395) was purchased from UPM biochemicals with an average molecular weight of 6,000 g/mol and less than 2% of ash content. Polylactic acid (PLA) pellets, namely, Inzea F38 were purchased from the Nurel biopolymers and contained 70% biobased content with a density of 1.23 g/cm^3^ and a melt flow index of 1.8 g/10 min and was measured with ISO 1133. Acetic anhydride (reagent grade, ≥99% purity) and Pyridine (reagent grade, anhydrous, 99.8%) were purchased from Sigma-Aldrich.

### 2.1 Lignin modification using acetylation

Kraft lignin (5 g, dry basis) was mixed with 15 mL of acetic anhydride and 50 mL of pyridine mixture (3:10 ratio). The reaction mixture was stirred at room temperature for 18 h. Then, the product was precipitated in ice-cold distilled water (1 L) and filtered through cellulose membrane filters. The precipitate was washed with distilled water several times to remove the unreacted reagents and dried in the oven at 40°C (Esakkimuthu et al., 2022b).

### 2.2 Lignin/PLA and modified lignin/PLA composites preparation

Lignin/PLA (LIG/PLA) and Acetylated lignin/PLA (aLIG/PLA) films were prepared using a solvent casting process using chloroform as a solvent. The total amount of dry substance in the composite films was kept constant at 2 g. PLA was used as a matrix, and different weight percentages (5%, 10%, and 20%) of lignin and acetylated lignin were added as fillers. PLA was stirred in 20 mL of chloroform at room temperature until complete dissolution followed by the addition of lignin and stirred for another 2 h before casting. The stirred solution mixtures were casted onto the 10 cm diameter glass Petri dish and dried at room temperature for 24 h. Then, the residual chloroform was removed using the vacuum drying process. The produced composite films resulted in a thickness of 200±50 µm. It is crucial to emphasize that we sourced PLA from Nurel Biopolymers and subsequently fabricated PLA films using a solvent casting process. Therefore, the term “Neat PLA” refers to the pure PLA obtained from the solvent casting procedure, without addition of any lignin materials.

### 2.3 Fourier transform infrared spectroscopy (FTIR)

FTIR analysis of kraft and acetylated kraft lignin was performed on a PerkinElmer spectrophotometer. The absorbance spectra of the samples were collected in the range of 4,000–400 cm^−1^ in the attenuated total reflectance mode and a total of 64 scans with a resolution of 4 cm^−1^ were collected in transmission mode.

### 2.4 Thermogravimetric analysis (TGA)

The thermal stability of LIG and aLIG samples were analyzed in Platinum pans using a Waters TA Instrument™ TGA 5500. The instrument was temperature calibrated using Nickel as calibrant. The samples (5–10 mg) were tested in a temperature range from 40°C to 800°C with a heating rate of 10°C min^-1^ under a nitrogen atmosphere with a flow rate of 25 mL min^-1^.

### 2.5 Differential scanning calorimetry (DSC) analysis

DSC measurements of LIG/PLA and aLIG/PLA biocomposites were performed with TA Waters Instruments, New Castle, DE, United States using Tzero Aluminum pans with Indium as reference material by applying cell constant/temperature calibration method under a nitrogen atmosphere with a temperature range of −25°C–210°C at a heating rate of 10°C min^-1^ with a flow rate of 25 mL min^-1^. Glass transition temperature (T_g_), and melting temperature (T_m_) were calculated from the recorded thermograms.

### 2.6 Mechanical test

The mechanical performance of neat PLA and PLA with LIG and aLIG composites was evaluated using a universal Testing Machine (Instron 4,204, United States) equipped with a 100 N load cell. Casted samples (5 mm width and 0.3 mm thickness) were tested according to the ISO 527–2 standard crosshead speed of 5 mm min^-1^ and an initial grip separation of 25 mm. Force and displacement were measured and recorded in the measurement environment of 50% RH at 23°C. Young’s modulus (E) was determined from the slope in the initial linear region of the stress-strain curve. A minimum of seven composite specimens were measured to calculate Young’s modulus, tensile strength, and elongation at break values.

### 2.7 Scanning electron microscopy (SEM)

The morphology of PLA-lignin composite specimens was examined using a field-emission scanning electron microscope (FE-SEM; Zeiss Sigma VP, Germany). Fractured surfaces from the tensile test were affixed to carbon tape to investigate surface morphological features. Subsequently, the samples underwent gold sputter-coating for 60 s at 10 mA. Micrographs of the surfaces were captured at an accelerating voltage of 5 kV using a secondary electron detector.

### 2.8 Molecular dynamics simulations

**FIGURE 1:**
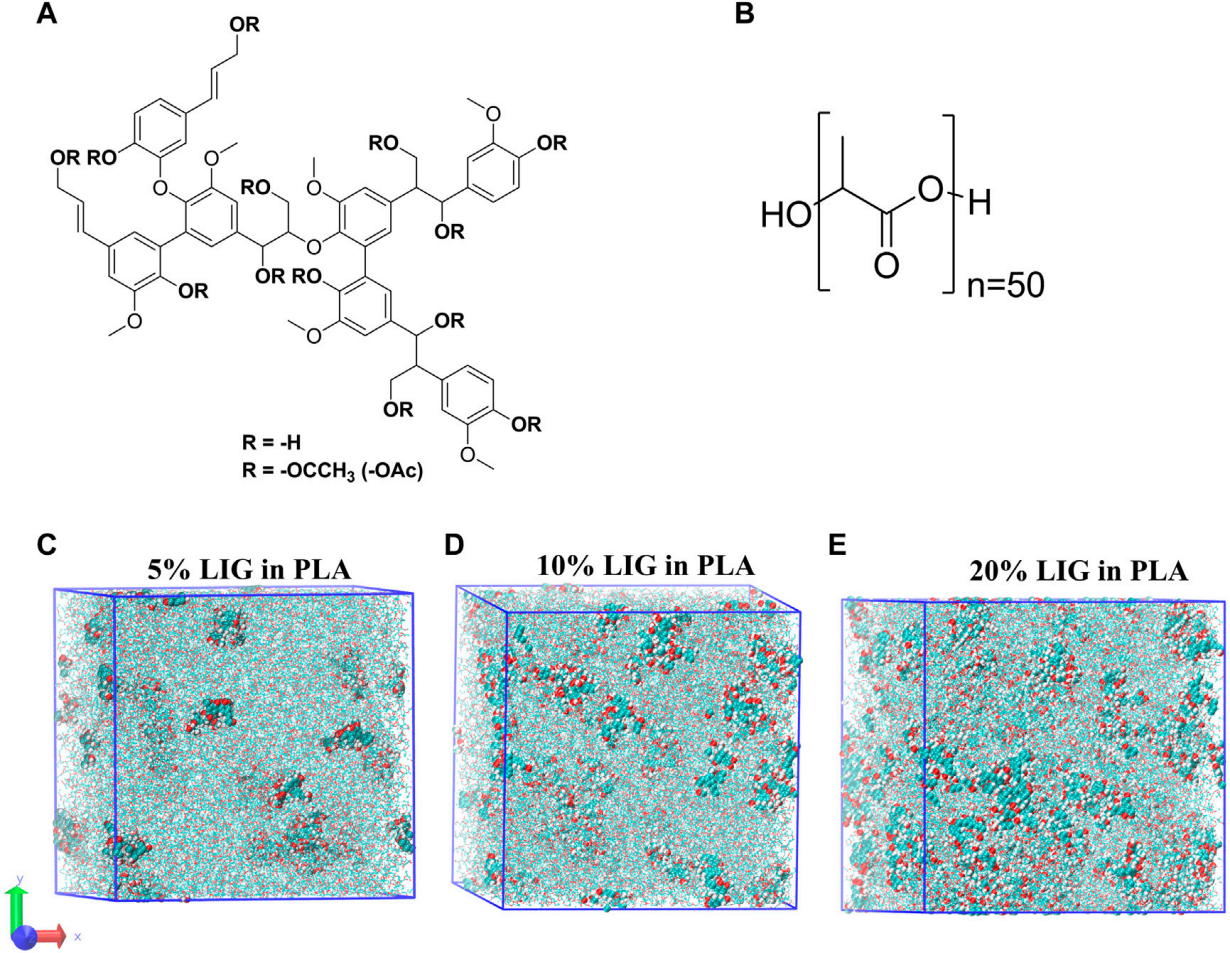
Molecular models used for MD calculations: **(A)** MD–Lignin model with acetyl (R = -OAc) substitution, **(B)** MD–PLA model with a degree of polymerization of 50, and **(C–E)** represent the equilibrated MD systems of lignin at 5%, 10% and 20% in PLA, respectively.

Due to its natural variability and heterogeneity at the molecular level, no universal structure for lignin exists to represent lignin for molecular dynamics. We have chosen a lignin model proposed by Vermass et al. (2019) which originally depicts softwood lignin. The selected model is shown in Figure 1C and the corresponding structure is composed of 6-G units and 1-P unit and contains 1 (β-O-4), 2 (5–5), 1 (4-O-5), and 2 (β −1) linkages with a total molecular weight of 1.2 kDa (unmodified lignin). In the case of acetylated lignin structure, the hydroxyl groups present in the phenyl rings and aliphatic chains were modified into corresponding acetylated lignin.

For MD simulations, the bonded and non-bonded (intermolecular) interactions are accounted by CHARMM force fields, and we used an excellent graphical interphase platform, CHARMM-GUI to build all lignin structures such as lignin (LIG) and acetylated lignin (aLIG) and PLA polymer for the simulations. In the case of PLA polymer, there are 50 repeating units or degrees of polymerization (DP = 50) used. No tacticity was considered in this study for PLA. Initially, separate simulation boxes were constructed for each system (LIG/PLA and aLIG/PLA) with three different weight percentages of lignin, such as 5%, 10%, and 20% with respect to PLA. The systems investigated in this study are presented in Table 1. It is important to validate the force field potential before performing extensive MD simulations to minimize the issues related to the representation of the systems. Therefore, we have conducted benchmark simulations for LIG and PLA materials separately and computed the density of the system from the simulations (at 298.15 K), and compared them with experimental data. The simulated density values for LIG and PLA were found to be 1,202 and 1,178 kg m^−3^, respectively. In the case of LIG system, various experimental density values are proposed (Ehrnrooth, 1984; Cotterill et al., 1996; Michelsen and Foss, 1996), ranging from 1,280 to 1,500 kg m^−3^. While for the PLA case, the experimental density is about 1,240 kg m^−3^ (Bhasney et al., 2019). These simulated values are consistent with experimental values and indicate that the employed potential is sufficient to represent the models. All MD simulations in this study were performed with GPU-supported GROMACS version of 2022.2 (Van Der Spoel et al., 2005; Abraham et al., 2015).

**TABLE 1:** The investigated systems containing the number of PLA, LIG, and aLIG with the corresponding composition, final density, and size of the simulation box (standard deviation in the parenthesis).

Systems	wt% of lignin	No. of lignin molecules	PLA	Final density (kg m^−3^)	Final box size (nm) x = y = z
**PLA**		0	255	1.178 (0.004)	10.9 (0.01)
**LIG/PLA**	5	42	255	1.181 (0.004)	11.1 (0.01)
	10	87	255	1.183 (0.003)	11.3 (0.01)
	20	198	255	1.188 (0.003)	11.7 (0.01)
**aLIG/PLA**	5	27	255	1.178 (0.003)	11.1 (0.01)
	10	58	255	1.179 (0.003)	11.3 (0.01)
	20	134	255	1.178 (0.002)	11.7 (0.01)

Each system presented in Table 1 was prepared with a cubic box size range from 17 to 20 nm based on the number of lignin molecules. For instance, the large box size of 20 nm was used as a starting dimension for 20 wt% of lignin. After randomly placing the lignin molecule in the simulation box, a linear PLA polymer configuration with DP = 50 was packed and followed by minimizing the box using the steepest descent algorithm to ensure no unfavorable impacts in the system. Equilibration simulation was carried out at 298.15 K for 3 ns in order to relax the entire system into a more appropriate state. The present work considered the random distribution of polymers such as lignin and PLA. Therefore, mixing the components toward attaining a highly mixed state was necessary to assure no void present in the simulation box. In such a case, a 3 ns run was performed at constant isotropic stress and temperature of 0.5 MPa and 600 K, respectively. The applied stress has been removed for the subsequent 3 ns run with 1 bar pressure at the same temperature of 600 K. After stress removal, there were two different simulations carried out, (i) cooling of the system from 600 K to 0 K to calculate glass transition temperature (T_g_) by decreasing the temperature at 0.1 K/ps and (ii) a production NPT (constant number of particles, constant pressure, and constant temperature) run of 15 ns at 298.15 K with 1 bar pressure. All the simulations were performed at 1 fs timestep, and pressure and temperature coupling were applied at every 1.0 ps and 2.0 ps, respectively. The pressure was controlled by the Parrinello-Rahman method. After production run calculations, the simulations were further continued by applying deformation with respect to three axes separately. Figures 1E–G depicts the simulation systems after the production run from 5%, 10%, and 20% of lignin in the PLA matrix. All the examined systems were deformed at a constant strain rate of 5 × 10^8^ s^-1^ with zero pressure and compressibility in a given axis under NPT conditions. The stress value of a system was calculated by averaging the stress value obtained from the x, y, and *z*-axis.

In this work, we have prepared PLA-matrix composites with softwood kraft lignin and its acetylated counterpart. The composite films prepared by solvent casting method were subjected to experimental characterization complemented with MD simulation studies. In the following, we first present the experimental results as a ground for reflecting the information obtained from the computational method.

## 3 Results

### 3.1 FT-IR

**FIGURE 2:**
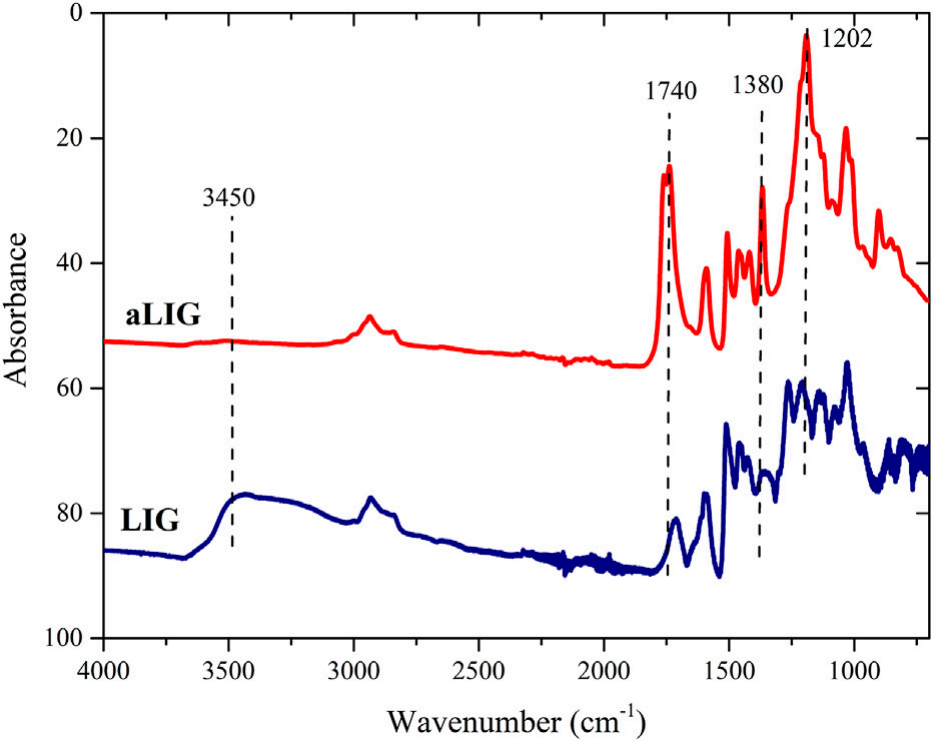
FT-IR spectra of crude Kraft lignin (LIG) and acetylated lignin (aLIG).

Since the fundamental approach in this work was to systematically compare softwood kraft lignin and corresponding acetylated lignin in PLA composites we decided to begin with validation of the conversion of the hydroxyl groups. The acetylation reaction of lignin was confirmed by FT-IR analysis and the spectra of unmodified lignin (LIG) and acetylated lignin (aLIG) are shown in Figure 2. We have mainly investigated the -OH stretching frequency peak observed for lignin at 3,450 cm^−1^ as it should diminish due to the acetylation. In the case of unmodified lignin, this particular peak exhibited a broad intensity whereas, after acetylation of lignin, it was almost flattened which indicated the substitution of hydroxyl groups of the lignin. Acetylated lignin showed the ester peak at 1,740 cm^−1^, C-H bond stretching peak at 1,380 cm^−1^ and acetyl group stretching peak at 1,202 cm^−1^ which are assigned to the non-conjugated carbonyl bonds of acetylated lignin. Absorbance changes were related to the degree of acetylation by measuring the height of the -OH stretching band at 3,300 cm^−1^ and normalized with the height of the C-C of aromatic skeletal vibration at 1,510 cm^−1^ which was taken as a reference (Saralegi et al., 2013; Gordobil et al., 2015). Hydroxyl group conversion (α) was calculated from Eq. 1, which provides the acetylation reaction achieved a conversion rate of 92%.

$$\alpha =1-\left[\frac{{\left(\frac{H_{OH}}{H_{ref}}\right)}_{t}}{{\left(\frac{H_{OH}}{H_{ref}}\right)}_{0}}\right]\times 100$$ (1)

### 3.2 Mechanical properties

**FIGURE 3:**
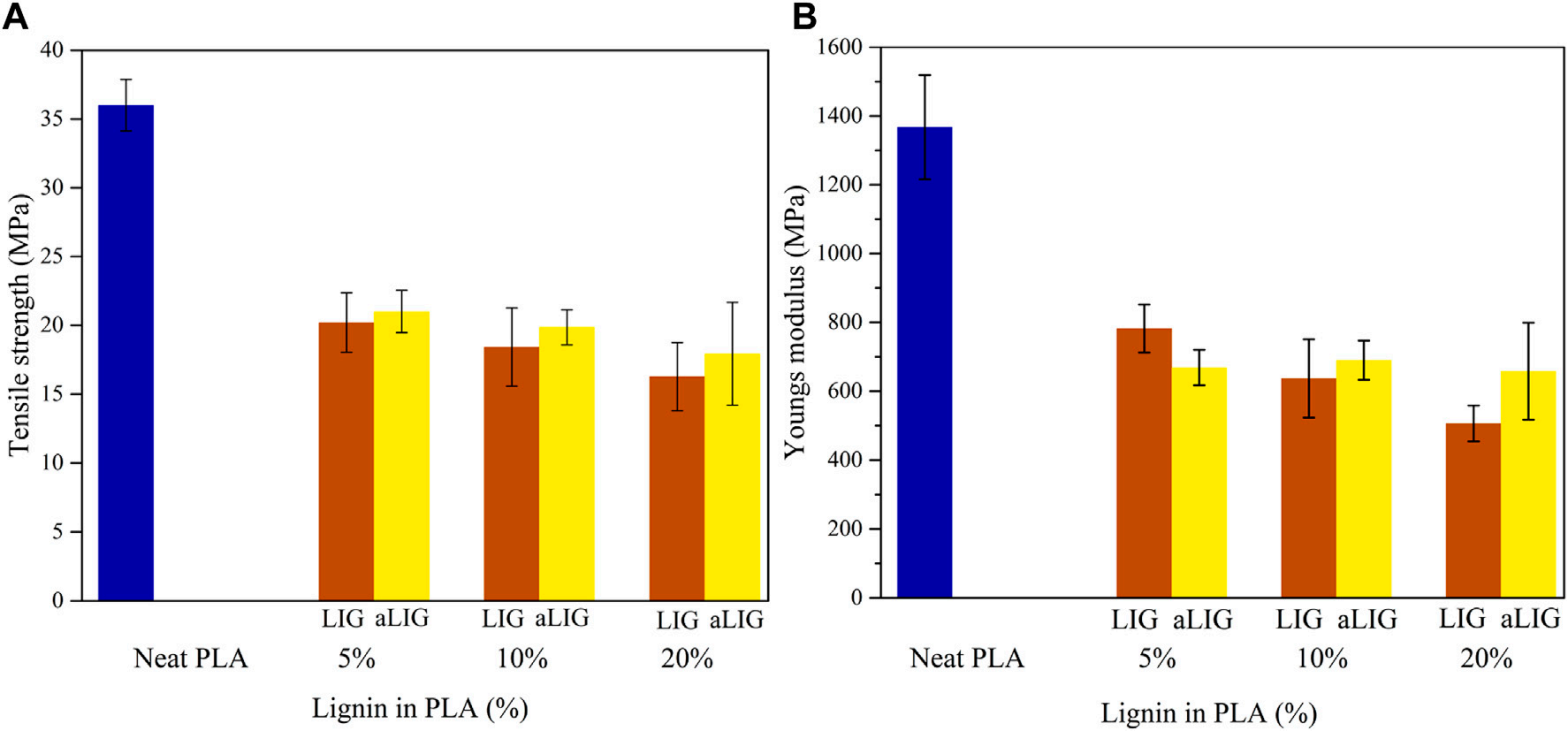
Mechanical properties of LIG/PLA and aLIG/PLA, **(A)** Tensile strength, and **(B)** Young’s modulus.

The presence of lignin in the PLA matrix can significantly alter its mechanical properties. We used tensile strength testing to obtain characteristic mechanical features of pristine PLA, LIG/PLA, and aLIG/PLA (Figure 3). In all the cases, pristine PLA showed better mechanical performance than the composites with lignin incorporated. It is interesting to see that the tensile strength and Young’s modulus were drastically reduced upon the addition of LIG and aLIG in PLA. We repeated the analysis and obtained the same results. The processing method employed for composites preparation (i.e., casting method) and plasticization effect might cause such significant changes in the mechanical properties. Comparing the composites such as LIG/PLA and aLIG/PLA showed that aLIG/PLA exhibited improved mechanical properties in terms of Young’s modulus and tensile strength. We hypothesize that the reason is that the unmodified lignin tends to aggregate in the dissolved PLA during the composite preparation, while acetylation reduces the aggregation effect and enhances the compatibility with the PLA. These findings are in line with prior observations by different authors (Gordobil et al., 2014; Kim et al., 2017).

### 3.3 Thermogravimetric analysis

**FIGURE 4:**
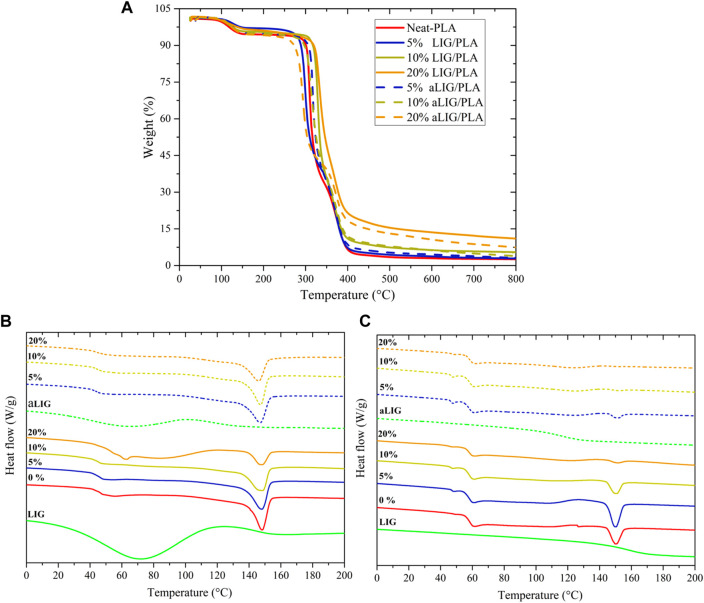
**(A)** Thermogravimetric curves of Neat PLA, LIG/PLA and aLIG/PLA, and **(B,C)** DSC scan curves of first heating and second heating scan: *Continuous lines*–PLA + *X* % of LIG and *Dashed lines–*PLA + *X* % of aLIG. (*X* is 5%, 10%, and 20%).

In addition to mechanical properties, another essential property of PLA composites is their thermal behavior which determines usability in many practical applications. Thermogravimetric analysis was performed to study the effect of lignin on the thermal degradation characteristics of PLA in the nitrogen atmosphere. Between 100°C and 150°C (Figure 4A), neat PLA lost around 5% of weight due to the residual amount of chloroform present from the casting process. Thermal degradation characteristics of neat PLA compared with the LIG/PLA and aLIG/PLA in Table 2. The results showed that increasing the weight proportion of kraft lignin systematically increased the thermal stability of the PLA composites. The 10% of LIG/PLA started to degrade at the higher temperature of 306°C compared to the neat PLA at around 293°C. There was a tradeoff between the thermal stability and lignin content of aLIG/PLA composites. At the lowest lignin content (5%), the thermal stability was up to 296°C while at the lignin content of 10% the thermal degradation onset temperature occurred earlier (270°C) than that of neat PLA (293°C). The results concluded that a lower weight percentage of derivatized lignin addition improved the thermal stability whereas, at a higher percentage, the possibility of lignin-lignin interaction was relatively high which results in a strong tendency to form aggregates. Therefore, it is crucial to understand the underlying reason behind the interaction between lignin-lignin and lignin-PLA in order to support the experimental findings and to further elucidate additional key properties. Such interaction mechanisms can be studied by a bottom-up approach, called computational modelling to investigate the materials at the atomic scale.

**TABLE 2:** Thermal (TGA) and DSC properties of neat PLA, LIG/PLA and aLIG/PLA composites. T_max_–Maximum temperature from TGA, and T_g_–glass transition temperature, and T_m_–melting temperatures from the first heating and second heating scan.

	TGA		DSC—First scan		DSC—Second scan	
	T_max_ (°C)	Residue at 800 °C (%)	T_g_ (°C)	T_m_ (°C)	T_g_ (°C)	T_m_ (°C)
Neat PLA	323	2.8	46.3	148.5	58.0	150.1
5% LIG/PLA	304	2.9	45.1	148.3	57.2	150.1
10% LIG/PLA	326	5.5	44.9	148.7	57.3	150.4
20% LIG/PLA	338	11.0	43.5	148.0	57.4	151.2
5% aLIG/PLA	322	1.7	44.1	147.4	57.3	151.3
10% aLIG/PLA	311	3.9	46.6	147.4	57.1	-
20% aLIG/PLA	291	7.5	44.9	146.4	58.1	-

### 3.4 Differential scanning calorimetry (DSC) analysis

DSC analysis was carried out to investigate the thermal characteristics of pristine PLA, lignin-PLA (LIG/PLA), and acetylated lignin-PLA (aLIG/PLA) (5, 10, and 20%) films. The obtained results from first and heating scans are shown in Figures 4B, C, and the glass transition temperature (T_g_) peak temperature (T_p_), and melting peak (T_m_) values are shown in Table 2. Kraft lignin showed a T_g_ value of 147.4°C, whereas acetylated lignin showed a T_g_ of 117.0°C. The difference in the T_g_ values of the underivatized and acetylated lignin could be due to the reason that unmodified lignin molecules are held together by strong intermolecular hydrogen bonding, van der Waals, and hydrophobic interactions, whereas the acetylation decreased the intermolecular forces between lignin molecules results in increased excluded volume and chain extension that would promote attractive interactions with PLA (Chung et al., 2013; Cachet et al., 2014; Gordobil et al., 2015).

Pure PLA cast films showed a T_g_ of 46.3°C and T_m_ of 148.5°C (Sharma et al., 2019), and the T_cc_ peak was absent with the addition of lignin and acetylated lignin, there was no significant change observed in T_g_. At a higher percentage of lignin addition, the T_g_ value shifted to a lower temperature which is attributed to increased molecular mobility of the chain induced by the plasticizing effect of the lignin. The cooling crystallization behavior of PLA disappeared with the addition of lignin and acetylated lignin. Also, a reduction in the T_m_ value was observed due to the amorphous characteristics of the lignin. The T_g_ values of all films increased during the second heating scan, and there was no significant difference observed between the films. Similarly, the T_m_ values followed a similar trend, with the melting temperature increasing during the second heating scan. It is also noted that in the cases of increased composition of aLIG in PLA (10% and 20% cases), no Tm peak was observed. The absence of a melting peak during the second scan can indicate that acetylation may lead to increased miscibility with PLA, suppressing and affecting the crystallization behavior.

### 3.5 Morphology analysis of the composite films

**FIGURE 5:**
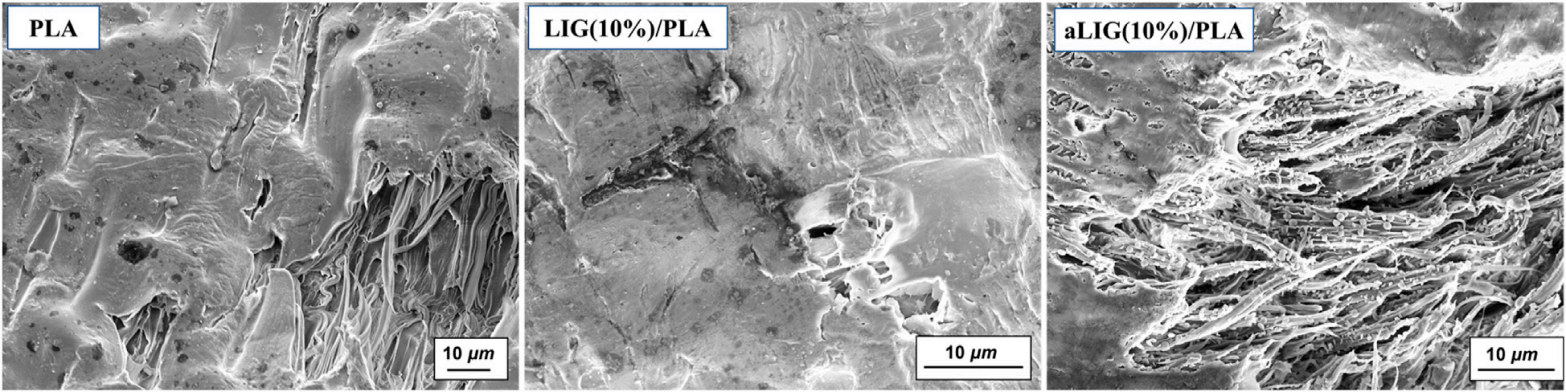
SEM micrographs of neat PLA, LIG/PLA (10%), and aLIG/PLA(10%).

Scanning electron microscopy (SEM) analysis provides the distribution of the lignin in the PLA polymer matrix and the tensile fractured LIG/PLA and aLIG/PLA specimens’ surface was analyzed. Neat PLA and a higher weight percentage (10%) of LIG and aLIG incorporated PLA are shown in Figure 5. The neat PLA surface was found to be smoother compared to the lignin-incorporated specimen (Figure 5). With 10% LIG addition results showed that the formation of voids and indication of a rough surface influenced Young’s modulus results. The tensile test results indicated that the addition of 10% of LIG in PLA decreased the tensile strength of the composite due to a lack of compatibility. On the other hand, the SEM image of 10% aLIG in PLA improved ductility (Figure 5). The small size particles of aLIG in the PLA acted as a plasticizer to hold together the PLA matrix and eventually improved the compatibility and enhanced the mechanical strength compared to unmodified lignin.

### 3.6 Molecular dynamics (MD) simulation results

#### 3.6.1 The prediction of glass transition temperature (T_g_)

**FIGURE 6:**
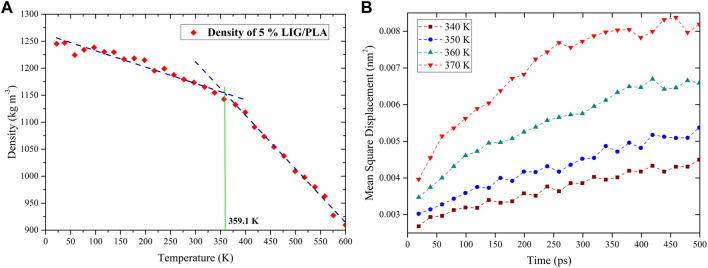
T_g_ calculation from, **(A)** linear fitting of the density-temperature curve, and **(B)** mean-square displacement simulation with different temperatures.

The glass transition temperature (T_g_) is an important thermal property of polymeric materials that describes the transition of amorphous polymer from the glassy state to the rubbery state. As such, the T_g_ also provides fundamental information on the thermal performance of the polymer system. T_g_ is usually calculated from the cooling stage, and thus we studied the cooling simulation from 600 to 0 K within 6 ns. During the simulation, the energy file was written every 1 ps and the density of PLA was calculated at each temperature. As shown in Figure 6A density values increase with decreasing temperature, and at a certain temperature value, the transition from a rubbery state to a glassy state (i.e., where T_g_ is calculated) can be seen. However, past research indicated that the T_g_ value could vary significantly concerning the density points from linear fitting. The authors performed T_g_ calculation for several compounds and obtained different T_g_ values for the same compounds by considering different fitting temperature points (Lin et al., 2021).

Nevertheless, the bulk of the literature points out that the T_g_ range can be calculated from the mean square displacement (MSD) calculations by performing additional MD simulations with different temperatures. With increasing temperature, the MSD gradually increases, but the MSD curve shifts drastically when the transition occurs in the system from a glassy state to a rubbery state due to the large motion of molecules in the rubbery state (Figure 6B). This is demonstrated by T_g_ which typically considered in the range between lower and higher temperature values. These MSD calculations greatly support the accurate prediction of the T_g_ range in the density calculation method. For instance, as shown in Figure 6B in the case of 5% LIG/PLA, the MSD values increase significantly from 350 to 360 K which indicates the range of glass transition temperature, T_g,_ and finally, the fitted density values show a final T_g_ value, around 359.61 K from Figure 6A. The fitted T_g_ values associated to pure PLA, LIG/PLA and aLIG/PLA composites using these two methods (density fitting and MSD calculation), are presented in Table 3.

**TABLE 3:** The glass transition temperature of polymer composites was obtained from the density fitting method and mean square displacement method.

	T_g_ by density fitting method (K)	T_g_ by MSD curve method (K)
Pure PLA	359.5	350–360
5% LIG/PLA	359.1	350–360
10% LIG/PLA	358.1	350–360
20% LIG/PLA	365.8	360–370
5% aLIG/PLA	350.5	340–350
10% aLIG/PLA	352.2	340–350
20% aLIG/PLA	364.3	350–360

From Table 3, the T_g_ value for pure PLA was found to be 359.5 K (∼86.4°C) and the addition of 5% LIG in the PLA matrix increases the T_g_ temperature of the composites. Nevertheless, the incorporation of 5% aLIG (acetylated) in the PLA matrix lowers the T_g_ value. The decreasing T_g_ value for aLIG/PLA composites indicated that the esterification reaction (acetylation) on the hydroxyl group substitution significantly reduces the hydrogen bonds and agglomeration, and leads to a lower T_g_ value compared to LIG/PLA. Increasing the wt% of both LIG and aLIG with PLA enhances the T_g_, of which 10% of aLIG almost exhibits a similar value to the T_g_ value of PLA. Finally, 20% of both modified and unmodified lignin showed higher T_g_ values. It should be noted that the calculated T_g_ values of all systems in Table 3 are overestimated compared to experimental values presented in Table 2. As the T_g_ is not a true first-order phase transition, this discrepancy cannot be ruled out due to different cooling conditions applied during experiments. Furthermore, a recent prediction from Lin et al., proposed T_g_ values for various amorphous systems and the obtained T_g_ value exhibited an error value of around 20.5°C. Therefore, considering the predicted value of pure PLA is 359.5 K (86.4°C) which is consistent with the values obtained from our experimental value of 46.29°C and literature values of around 60°C by taking into account with the error, around 20.5°C (Kim et al., 2017; Esakkimuthu et al., 2022a). Moreover, the trend observed from the simulation for all T_g_ values associated with the different wt% can be correlated directly to the experiments.

#### 3.6.2 Fractional free volume

**FIGURE 7:**
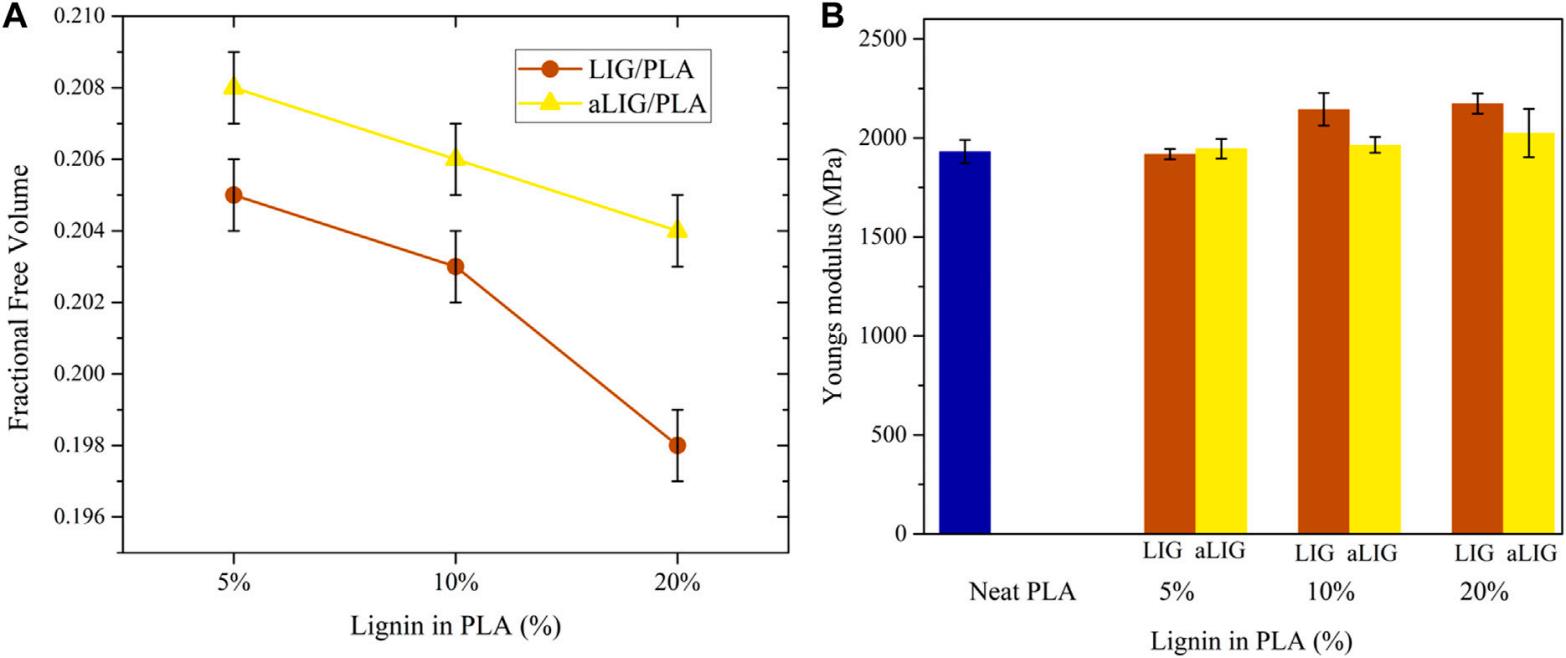
**(A)** The fractional free volume (FFV) of LIG/PLA and aLIG/PLA, and **(B)** The Youngs modulus values of LIG/PLA and aLIG/PLA based composites with different weight %.

The fractional free volume (FFV) defines the intermolecular space dispersed in the system, like in the form of an empty area or hole to enable the molecules’ motion. The FFV is normally calculated from the relation of occupied volume V_0_ and free volume V_f_, and therefore, FFV = V_f_/(V_0_ + V_f_). The FFV is calculated by inserting a probe radius of 0 nm and the determined FFV is known as true free volume. The obtained FFV results shown in Figure 7A indicated that all LIG/PLA composites exhibited lower FFV than compared corresponding aLIG/PLA systems. The inclusion of an acetyl side chain in the hydroxyl group is expected to increase the distance between PLA and acetylated lignin polymers. The higher content of LIG (20%) in PLA presented the lowest FFV, which indicated that the motion of PLA is majorly restricted due to hydroxyl groups present in LIG polymer.

#### 3.6.3 Mechanical properties: Young’s modulus

Young’s modulus is used to account for the elastic mechanical properties of LIG/PLA and aLIG/PLA composites. As described in the method section, a constant strain rate of 5 × 10^8^ s^−1^ was applied in the model system and we performed an elongation up to 20% of the original length, in which an initial 4% of strain was taken into account to determine Young’s modulus. The calculated Young’s modulus values correspond to the average of three axes (x, y and z) due to the fact of heterogeneity of composites. The average Young’s modulus was calculated, and the results are shown in Figure 7B. The addition of low wt% (5%) of LIG and aLIG in the PLA matrix resulted in a similar Young’s modulus as for neat PLA, and increasing the wt% of both LIG and aLIG increases the modulus values. The Young’s modulus obtained from MD simulations was overestimated, about 41.1% for PLA than experimental values (shown in Figure 3B). Various reasons can be associated with such a difference, including the strain rate applied in MD, the polymer chain lengths considered for the MD study and the composite processing techniques used for PLA synthesis. On the other hand, the MD method precited Young’s modulus for LIG/PLA and aLIG/PLA composites showed almost three times higher than the values obtained from experiments. The mechanical properties calculation with the MD method indicated that the incorporation of lignin or acetylated lignin showed a minor impact on Young’s modulus, while experimental data presented in Figure 3, showed a clear difference between various composites.

#### 3.6.4 Interaction energy

**FIGURE 8:**
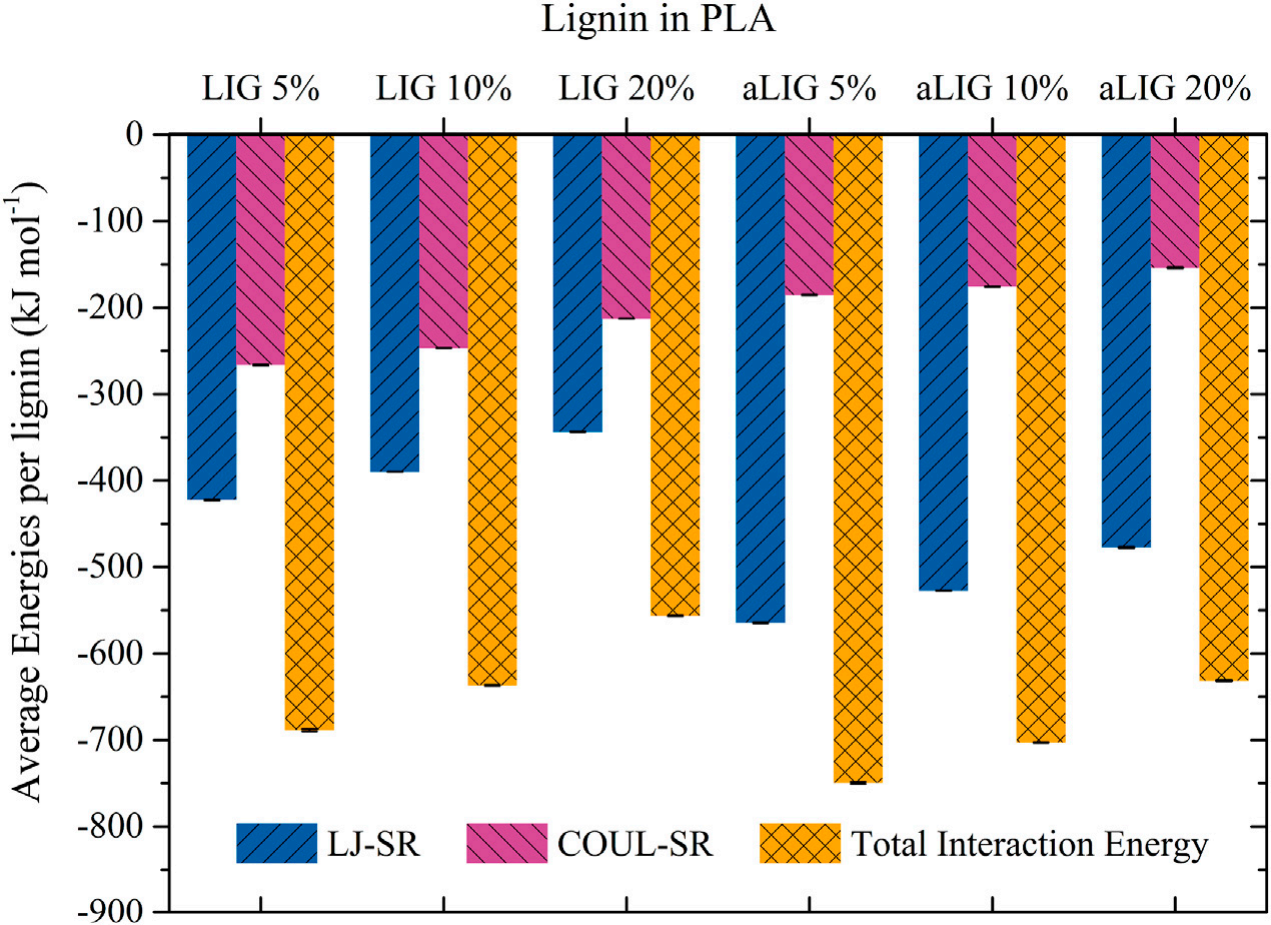
The Lennard-Jones (van der Waals) energy, Coulombic energy, and total interaction energies of LIG and aLIG with PLA at different compositions.

A short-range interaction between lignin and PLA polymers was calculated to quantify the binding interaction by taking van der Waals interaction (Lennard-Jones: LJ) and coulomb interaction energy terms. These energy values were averaged with respect to one lignin (LIG and aLIG) to compare both systems. The obtained results shown in Figure 8 revealed that the total interaction energy of aLIG exceeded that of unmodified lignin (LIG). However, it is interesting to see that Lennard-Jones short range interaction energies were higher in the case of aLIG/PLA contributing to the elevated total interaction energies of acetylated lignins with PLA. On the other hand, Coulomb energy was more dominant in the case of LIG/PLA than aLIG/PLA. It is indicated that the hydroxyl groups present in LIG majorly increases the binding with PLA polymer, which significantly decreases upon modifying the hydroxyl groups with acetylation.

#### 3.6.5 Hydrogen bond analysis

**FIGURE 9:**
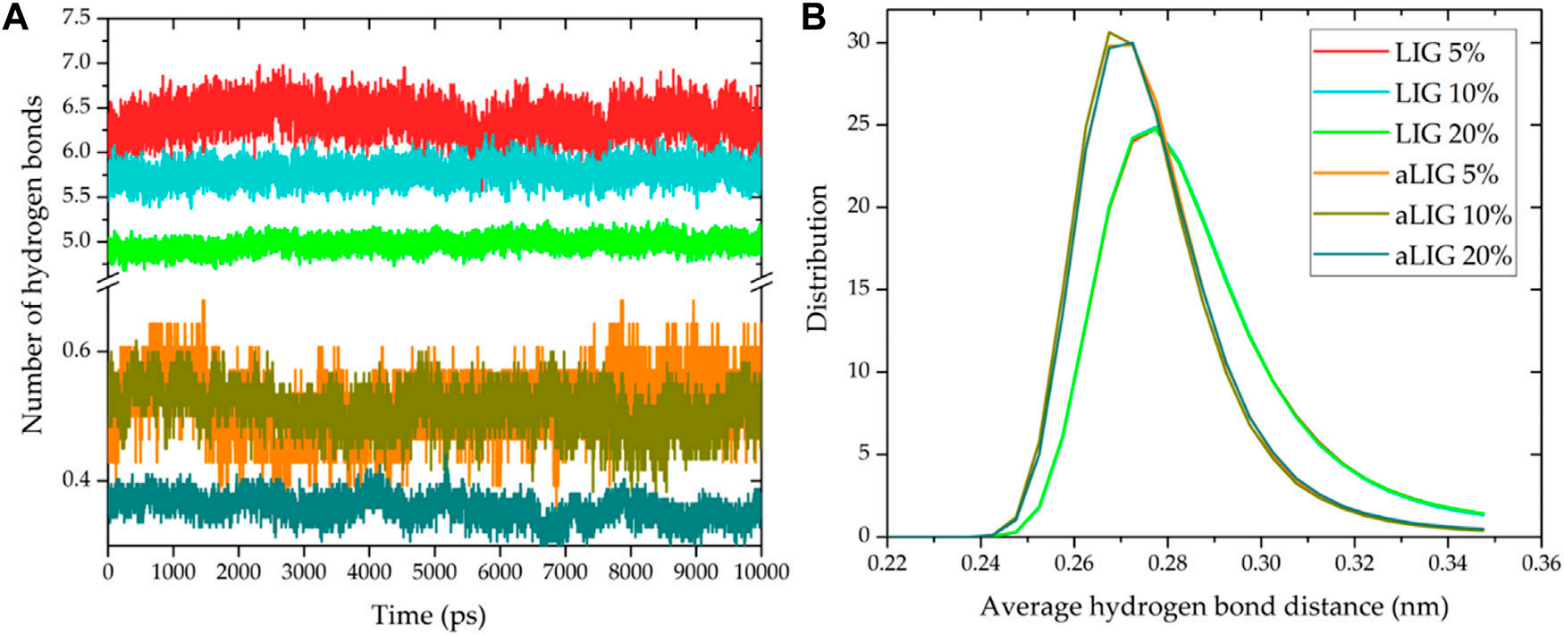
Hydrogen bonding characteristics of LIG/PLA and aLIG/PLA systems, **(A)** the average number of hydrogen bonds **(B)** the distance distribution of hydrogen bond formation.

The average number of hydrogen bonds between LIG/PLA and aLIG/PLA was calculated by considering the criteria of the cut-off distance of 3.5 Å and an angle of 30°, and the results are presented in Figure 9. In the case of LIG/PLA systems, the average number of H-bonds was significantly higher compared to aLIG/PLA systems which is due to the fact that aLIG composites contain only the carboxylic acids as donor groups. In contrast, the abundant hydroxyl groups present in the unmodified LIG molecules possess both donor (-H) and acceptor (-O). Therefore, LIG tends to form a higher number of hydrogen bonds, with PLA and acetylation, markedly reduces the possibility of hydrogen bonding with PLA. At higher lignin content the number of hydrogen bonds in both LIG/PLA and aLIG/PLA decreases as the combination of LIG-LIG and aLIG-aLIG increases other intermolecular interactions at the cost of hydrogen bonding. The distance associated with the hydrogen bond formation was evaluated, and the distance distribution is shown in Figure 9B. The acetyl groups in aLIG tend to make a shorter hydrogen bond (about 0.268 nm or 2.68 Å) than LIG/PLA composites, about 0.28 nm or 2.8 Å, which may be due to the acetylation reduced ionic repulsion between the lignin molecules the lignin polymer.

#### 3.6.6 Radial distribution function

**FIGURE 10:**
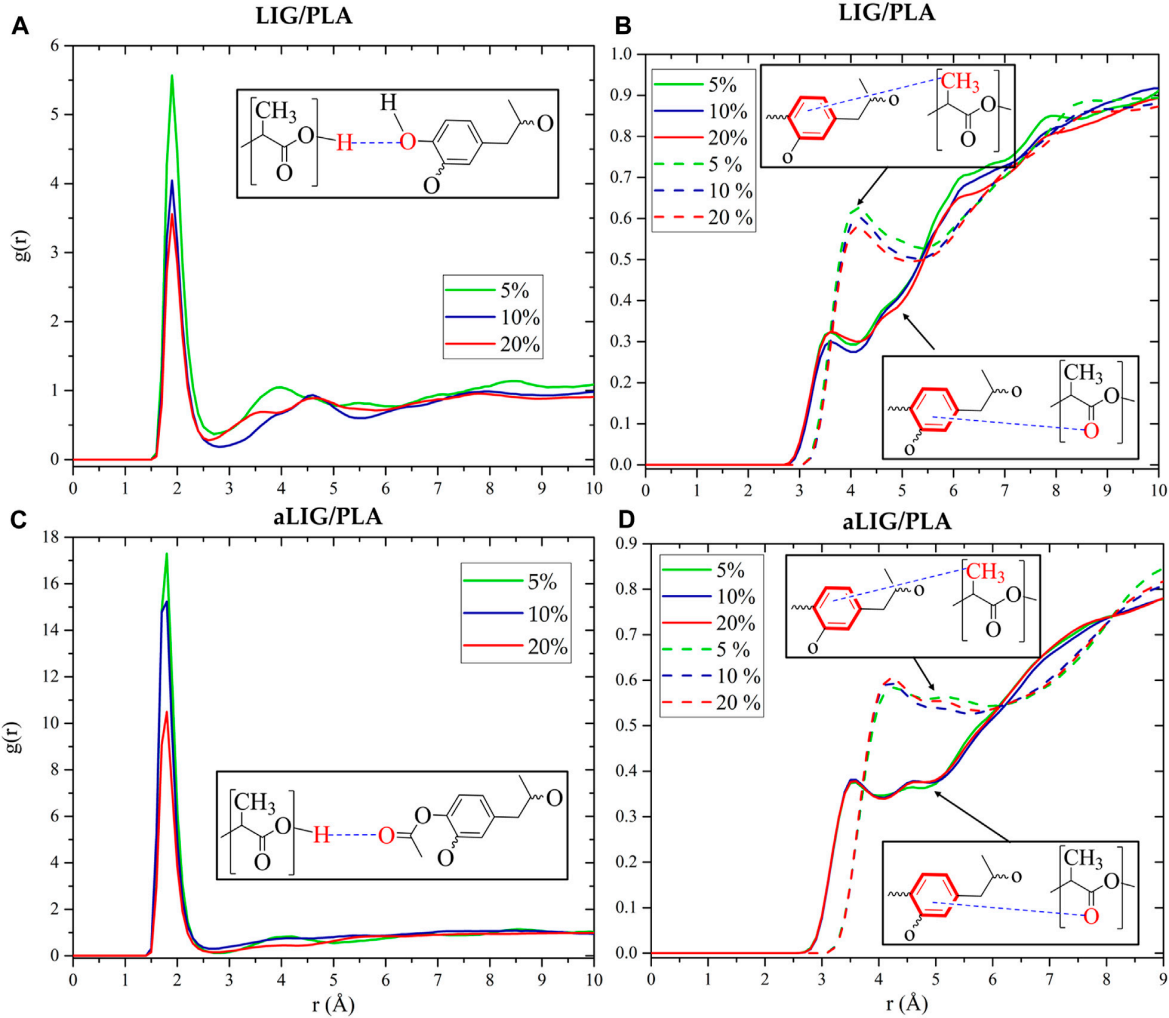
Radial distribution function between different atoms in LIG/PLA **(A,B)** systems, and aLIG/PLA **(C,D)** systems.

The radial distribution function (RDF) provides a detailed insight into understanding the molecular arrangements of atoms or molecules in the system. The RDFs of LIG/PLA and aLIG/PLA were calculated and shown in Figure 10. As seen in Figure 10, the typical distance between oxygen in unmodified lignin (LIG) and hydrogen attached to oxygen in PLA was found to be 1.9 Å. The g(r) distribution decreased at higher content of LIG in PLA due to the increasing contacts of LIG-LIG that dominated over those of LIG-PLA. Similarly, the average distance of the center of mass of aromatic rings (Ar) with carbonyl oxygen (O=C) and methyl group (CH_3_) in PLA was calculated, and the results showed that Ar-O_C_ exhibited a shorter distance than Ar-CH_3_, due to the fact of strong interaction of polar groups (O=C) than non-polar group (CH_3_). In the case of aLIG/PLA (Figures 10C, D), the calculated distance distribution between the carbonyl oxygen from the acetyl group in lignin and the hydroxyl group in PLA exhibited a similar distance, about 1.8 Å in all examined weight percentages. This correlation indicated that acetylation of lignin not only increases the chain length but also reduces the distance of interaction with hydroxyl groups in PLA compared to the case of LIG/PLA, thereby enhancing compatibility. The distance between the aromatic ring and carbonyl oxygen in PLA averaged 3.6 Å. In the case of aromatic ring–methyl group, the average distance was 4.2 Å. Changing the weight % in aLIG/PLA was found to have a minimal effect on the hydrogen bonding distance.

## 4 Discussion

Our combined experimental characterization and computational modelling results have provided new insights into the compatibility of lignin in polylactic acid composites. The the experimental results indicated that acetylation may enhance the plasticity and motion of acetylated lignin in PLA, which moderately decreases the glass transition temperature.

Molecular dynamics (MD) simulations overestimated the glass transition temperatures and Young’s moduli for all systems studied due to their sensitivity to various parameters and fitting methods employed. In contrast to the modelling results, indicating no marked changes in Youngs’s moduli, our experimental results showed that the addition of lignin or acetylated lignin to PLA leads to a drastic decrement in Young’s moduli of the composites.

We obtained a Young’s modulus value of 1,400 MPa for PLA, which is lower than our previously proposed value of approximately 2,100 MPa (Esakkimuthu et al., 2022a). It has been noted that the mechanical properties of composites are significantly influenced by the processing methods employed during fabrication. In this study, the solvent casting method was used, which tends to result in lower tensile strength and Young’s moduli compared to other methods such as extrusion molding, injection molding, and compression molding (Raj et al., 2021). Surprisingly, both the tensile strength and Young’s moduli values of the composites decreased to one half of those of PLA, contradicting values reported in the literature (Kim et al., 2017). This drastic change could be attributed to the plasticization effect and inferior compatibility of lignin and its counterpart with PLA. We observed a lower Tg (46.3°C) value in the present study compared to our previously reported value of 60.3°C (Esakkimuthu et al., 2022a). The differences in thermal properties between the neat PLA and LIG/PLA composite obtained in current study by solvent casting method could be attributed to several factors, including, (i) process method, in which extrusion method is conducted at high-temperature and high shear conditions, (ii) orientation of polymer chains in the composites, in which solvent casting exhibited lower degree of molecular orientation than extrusion, and (iii) Solvent casting, being a milder process, might preserve the chemical structure better than extrusion method which may induce higher possible chemical interactions or reactions. However, our results for the predicted glass transition temperatures of both LIG/PLA and aLIG/PLA are consistent and show a similar trend compared to earlier results (Kim et al., 2017). It should be noted that incorporating a higher weight percentage of lignin (40%) in PLA resulted in increased brittleness, making it impractical to consider the corresponding mechanical properties in this study.

It is essential to highlight additional properties wherein acetylated lignin demonstrates notable enhancements, including improved water permeability, surface wettability, and water solubility. As reported by Kim et al. (2017), the combination of acetylated lignin with PLA exhibited a lower water vapor transmission rate compared to unmodified lignin. Similarly, Johansson et al. (2023) observed a significant delay in moisture absorbance for acetylated lignin over 28 days, and Kim et al. (2023) noted the maintenance of contact angles from water droplet experiments. The acetylation reaction primarily blocks hydroxyl groups in lignin, increasing hydrophobicity and consequently reducing lignin’s interaction with water.

MD simulations conducted at the bulk scale revealed that aLIG/PLA-based composites exhibited a larger free volume compared to LIG/PLA, indicating that the increased side chains in lignin enhance plasticity. Similarly, interaction energy calculations demonstrated that electrostatic interactions played a significant role in LIG, likely due to the presence of hydroxyl groups, while such interactions were lower in aLIG/PLA systems. The modification of lignin with acetylation led to a significant reduction in the number of hydrogen bonds, which may explain the decreased aggregation of modified lignin compared to unmodified lignin. Therefore, the comprehensive analyses including the hydrogen bonding, interaction energy and free volume prediction by MD elucidated the underlying origin of intermolecular forces within the composite system. The results associated to MD are in good agreement with the experimental results of this work.

It is noted that the Young’s modulus obtained from the MD simulations aligns with previous literature results where composites were prepared using the extrusion method (Gordobil et al., 2014; Esakkimuthu et al., 2022a). There are disparities between the mechanical properties obtained from MD simulations and experimental values in the present study. Such differences can be attributed to several factors, such as: (i) MD simulations operate at the molecular scale and on nanosecond timescales; in contrast, experimental testing measures macroscopic properties over longer durations, (ii) MD simulations depend on the reliability of force fields and parameters used. The limitations in predicting dynamic and complex behaviors, especially arising from structurally heterogeneous lignin molecules, contribute to differences between simulated and experimental outcomes, (iii) experiments consider various external factors that might not be fully accounted for in simulations, such as impurities, processing conditions, and structural variations, and (iv) aggregations in polymer composites may occur on longer time scales, posing challenges in capturing them within the simulation timeframe. However, it is important to acknowledge that no aggregation was observed during the MD simulations, as the lignin molecules were randomly distributed within the PLA matrix. In contrast, experimental processes may exhibit apparent aggregation. Furthermore, the present MD simulation focused on investigating the interfacial interactions between lignin and PLA, as they predominantly influence the properties of the composites.

## 5 Conclusion

Intermolecular interactions play a vital role in lignin-lignin and lignin-PLA systems, and understanding these interactions is crucial for utilizing composites in various value-added applications, including packaging. Therefore, this study employed a combined approach of experimental and computational modeling to elucidate the fundamental properties of lignin/PLA composites. Solvent-casted lignin/PLA and acetylated lignin/PLA composites with different weight percentages of lignin (5%, 10%, and 20%) were investigated. Mechanical characterization revealed that acetylation of lignin improved both the tensile strength and Young’s modulus of the corresponding composites. The glass transition temperature of the composites moderately decreased with 5% and 10% weight fraction of acetylated lignin, whereas no significant effect was observed with the unmodified lignin at similar lignin contents. To investigate the underlying reasons for these differences, computational modeling methods such as molecular dynamics (MD) were employed and compared with experimental results. The MD results indicated that acetylation of lignin decreases the T_g_ and Young’s modulus of aLIG/PLA compared to LIG/PLA. However, the obtained MD results for T_g_ and Young’s modulus of pure PLA, LIG/PLA, and aLIG/PLA were found to be overestimated compared to experimental findings. The interaction energy results showed that aLIG exhibited higher van der Waals forces and lower Columbic interaction with PLA compared to unmodified lignin. Additionally, a higher fractional free volume was observed for aLIG. One possible reason is that acetylated lignin significantly reduces the number of hydrogen bonds, which are central to understanding the aggregation of lignin in the PLA matrix. Overall, this combined study involving experiments and computational modeling provided new insights into the compatibility of lignin with PLA, which can be further enhanced by tailoring modifications for PLA-based composite materials. However, further studies are suggested to evaluate the contribution of hydrophobic interactions to the total energy of interaction between esterified lignin and PLA.

## Data Availability

The raw data supporting the conclusions of this article will be made available by the authors, without undue reservation.
